# Patternable and Widely Colour-Tunable Elastomer-Based Electroluminescent Devices

**DOI:** 10.1038/s41598-018-21726-x

**Published:** 2018-02-20

**Authors:** Seongkyu Song, Hyunseok Shim, Sang Kyoo Lim, Soon Moon Jeong

**Affiliations:** 1 Smart Textile Convergence Research Group, DGIST, Daegu, 42988 Republic of Korea; 2 Intelligent Devices and Systems Research Group, DGIST, Daegu, 42988 Republic of Korea

## Abstract

We demonstrate wide colour tunability of polydimethylsiloxane-based alternating-current-driven electroluminescent devices with intrinsically stretchable characteristics achieved by simply modulating the electrical frequency. By employing both a screen-printed emitting layer and frequency-dependent colour tuning of ZnS:Cu-based phosphors, we demonstrate various coloured patterned images in a single device. We also show enhanced colour-tuning performance by mixing multi-colour phosphors, which results in a broad range of available coordinates in colour space. We believe that our demonstrated method could be used for manipulating broader colour expression as well as in various applications involving stretchable devices.

## Introduction

Recently, significant progress has been made in the development of new techniques for stretchable electronics, overcoming the limitations of rigidity and brittleness of conventional devices. To achieve such stretchability in inorganic electronics, semiconductor nanomaterials configured into wave-like shapes have been intensively studied. Such techniques are expected to be promising for partially stretchable structures employing either intrinsically stretchable materials or novel device configurations^1–3^.

Polydimethylsiloxane (PDMS)-supported zinc sulfide (ZnS) composites (PDMS + ZnS) sandwiched between a pair of mechanically compliant electrodes (e.g. silver nanowires; AgNWs) have been studied as an alternating-current-driven electroluminescent device (ACEL) because of its unique characteristics of intrinsically stretchable electroluminescence (EL)^4–11^, and mechanoluminescence (ML)^11–18^. Such PDMS composites embedded with luminescent inorganic phosphors are very attractive due to their simple fabrication process, mechanical robustness, chemical inertness, low cost, and sufficient pliability to be fully stretchable even under application of an electric field. Various studies of PDMS-based ACEL devices have focused on improving the brightness^7^, stretchability^8,9^, and range of colours^10^. The inherent excellent mechanical conformability of the composite allows it to withstand external deformations without loss of functionality, leading to unprecedented applications, including wearable multifunctional sensors/displays, biomedical devices, and electronic skin, which are subject to a wide range of body movements^5,19^.

To make a stretchable emitting layer (EML), PDMS is mixed with luminescent materials. The most common material used for stretchable ACEL devices is ZnS doped with metallic elements (such as Cu, Al, and Mn) creating luminescent centres. In general, the luminescence from ZnS is produced either by introducing lattice defects that deviate from the stoichiometric Zn/S ratio or by doping with impurity atoms, which are called activators^20^. Generally, a luminescent material should emit a single colour (i.e. have a narrow emission spectrum at specific wavelength). However, in our previous study, we reported various colours with different emission wavelength ranges obtained by modulating electrical frequencies in Cu-doped ZnS phosphors^13,15^. At high electrical frequencies, blue EL occurs because of the higher contribution from the deepest energy levels (blue centres). In contrast, at low frequencies, the emission is dominated by energy levels emitting in the green wavelength range.

This frequency-dependent emission can be explained by donor-acceptor (D-A) pair emission from the ZnS phosphors doped with Ib elements (activator) and IIIb or VIIb elements (co-activator). To date, the most well-known phosphors are the green-emitting ZnS:Cu,Al and ZnS:Cu,Cl^21–25^. Luminescence takes place in the centres of pairs of donors (co-activators) and acceptors (activators) associated at the second and third nearest-neighbour site, and luminescence transition occurs from the excited state of donors to the ground state of acceptors. The emission peak shifts toward higher energy (i.e., shorter wavelengths) with increasing excitation intensity based on D-A pair emission theory^21,22^. We noted that this colour-tunable property could be used for producing colourful images in stretchable devices by simply modulating frequencies if an EML could be patterned. Furthermore, from the viewpoint of light-emitting applications, patterning of emitting regions is highly attractive to form desired images. However, no previous work has been reported regarding such patterning of EMLs.

Here, we demonstrate a patterned, widely colour-tunable elastomer-based ACEL device with intrinsically stretchable characteristics achieved by employing a screen-printed EML. Screen-printing methods have been widely adopted due to the advantages of fast fabrication and low cost. By combining this process with colour tunability, we demonstrate patterned images emitting different colours in a single device by simply modulating the electrical frequency. Such a method is expected to be applicable for broader colour expression in future ACEL applications.

## Results and Discussion

**Figure 1:**
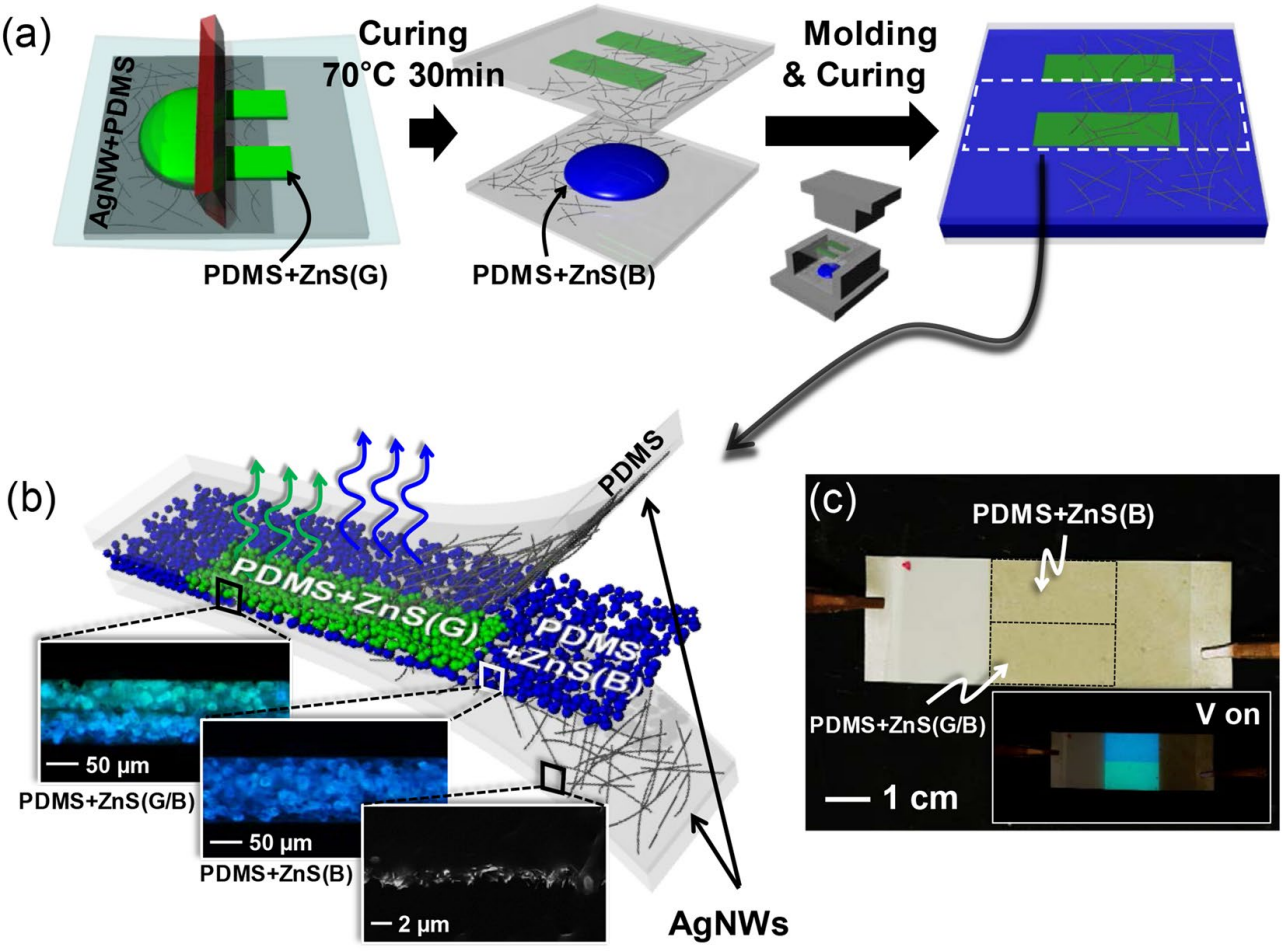
(**a**) Schematic diagram showing the ACEL device fabrication. (**b**) Schematic illustration of the ACEL device structure. Insets: optical and SEM images showing cross-sections of PDMS + ZnS(G/B), PDMS + ZnS(B), and AgNWs embedded in PDMS. The photos were taken under UV (365 nm) irradiation to visualize each layer. (**c**) Optical image of the ACEL device. Inset shows light-emitting ACEL under 400 Vpp and 1 kHz.

To create a PDMS + ZnS mixture emitting different colours, two commercially available ZnS:Cu-based phosphors emitting blue and green EL in a moderate frequency range (~1 kHz) were employed, hereinafter referred to as ZnS(B) and ZnS(G), respectively. These two phosphors displayed different colour tendencies in response to electrical frequencies. To fabricate an ACEL device with multi-coloured EL, a screen-printing method was used to pattern the PDMS + ZnS(G) mixture. Figure 1a shows a simplified schematic diagram of the fabrication process of the patterned ACEL device. Briefly, a PDMS + ZnS(B) mixture was sandwiched between two AgNW-embedded PDMS plates; one of the plates contained cured PDMS + ZnS(G) stripes. The detailed fabrication process is presented in the Methods section and Fig. S1 (Supplementary Information). After moulding and curing, the device partially contained a bilayer-structured patterned PDMS + ZnS(G) on a PDMS + ZnS(B) layer, which is hereinafter referred to as PDMS + ZnS(G/B) (Fig. 1b). Although one side of the substrate was coated with PDMS + ZnS(G), we note that a uniform thickness (approximately 100 µm) could be obtained over the entire sample area, including PDMS + ZnS(G/B) and PDMS + ZnS(B), as the viscous PDMS + ZnS(B) mixture under the printed layer spread during the moulding process (see inset images in Fig. 1b). This entirely uniform thickness enables the creation of a film-type stretchable device emitting uniform light from PDMS + ZnS(G/B) and PDMS + ZnS(B) sandwiched by stretchable AgNW-embedded PDMS plates (Fig. 1c).

**Figure 2:**
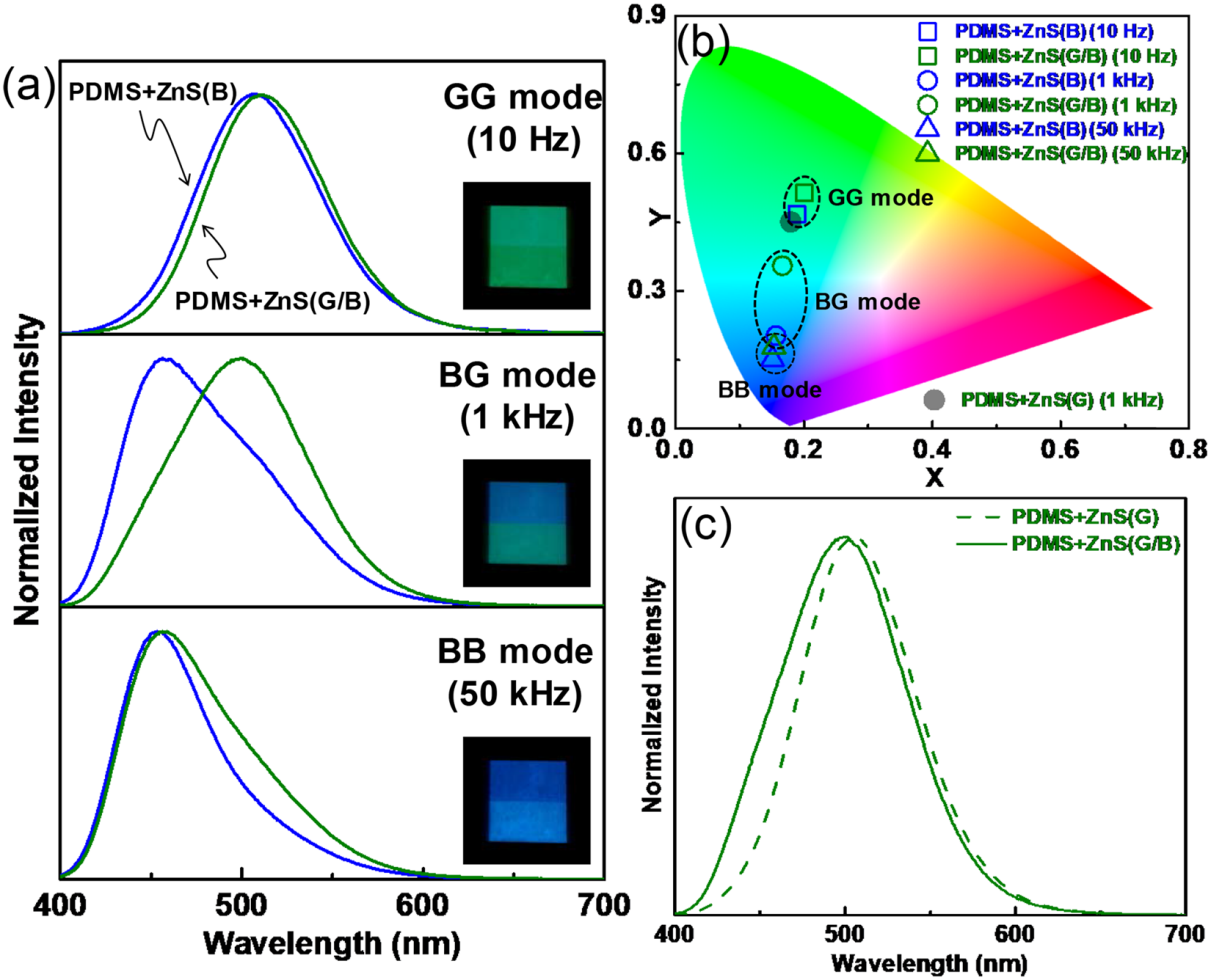
(**a**) EL spectra (400 Vpp) from PDMS + ZnS(B) and PDMS + ZnS(G/B) under different frequencies (10 Hz, 1 kHz, and 50 kHz). Insets: EL images. (**b**) CIE coordinates (*x, y*) calculated from the EL spectra. (**c**) Normalized EL spectra from PDMS + ZnS(G/B) and PDMS + ZnS(G) ACEL devices (400 Vpp; 1 kHz).

The aim of this study was to use electrical frequency modulation-induced colour tuning to create various coloured patterned images in an ACEL device. To achieve this, it was necessary the measure the EL colour difference between PDMS + ZnS(G/B) and PDMS + ZnS(B), which eventually creates the patterned image in the device. Figure 2a shows the frequency dependence of the EL spectra from each region. Here, we selected representative frequency conditions for preliminary experiments to highlight the largest colour difference. At a frequency of 10 Hz, both PDMS + ZnS(G/B) and PDMS + ZnS(B) emitted green EL spectra with similar peak positions, 511 nm and 507 nm, respectively. However, at 1 kHz, the EL peak position from PDMS + ZnS(B) shifted to a shorter wavelength, resulting in a blue colour, whereas PDMS + ZnS(G/B) still emitted green luminescence (a similar colour to that produced at the lower frequency). This colour difference helps visualize the patterned image. When the electrical frequency increased to 50 kHz, the luminescence from PDMS + ZnS(G/B) also shifted to blue, much like PDMS + ZnS(B). Thus, the outputs could be easily categorized as there were three colour regions within a given sample consisting of the ZnS(B) and screen-printed ZnS(G/B) layers: (1) green-green (GG mode; low frequency); (2) blue-green (BG mode; medium frequency); and (3) blue-blue (BB mode; high frequency) modes. These properties can be visualized as CIE coordinates in colour space and grouped into three regions (GG, BG, and BB modes) simply by increasing the electrical frequency from 10 Hz to 50 kHz (Fig. 2b). This colour dependence on frequency is a basic principle of the present work that allows the production of images by simply modulating the frequency.

Although the explanation for powder-based ACEL is still open to speculation, the most popular and reasonable theory for EL excitation in Cu-activated ZnS phosphors is the bipolar field-emission model proposed by Fischer^26–28^. First, the EL emission from ZnS:Cu is explained by assuming conducting Cu-decorated needles existed in ZnS particles. In fact, the fabrication of ZnS:Cu particles requires high-temperature firing process. The presence of conducting needles is caused by the precipitation of Cu during cooling. This reduction of Cu solubility in ZnS results in embedded conducting Cu_x_S needles in ZnS. These conducting Cu_x_S needles concentrate the applied electric field at their tips, which becomes strong enough to induce tunnelling of holes from the centres. Then, upon reversal of the fields, the emitted electrons recombine with trapped holes to produce light. Thus comet-shaped EL emission occurs along the Cu_x_S precipitates. At high frequency, the comet-shaped emitting lines became sharp and blue emission predominates, whereas the emitting lines were spread out and the green emission was dominant at low frequency^27^.

To explain the GG, BG, and GG modes, it is necessary to understand the colour change behaviour in Cu-doped ZnS resulting from modulating the electrical frequencies. First, the luminescence mechanism should be considered to elucidate the colour changes in more detail. As noted in the previous section, the emission from ZnS phosphors is governed by D-A transitions from donor (co-activator)-acceptor (activator) pairs. The emitted photon energy depends on the spatial separation of these elements and is expressed as E(R) = E_G_ − (E_A + _E_D_) + e^2^/KR, where E(R) is the emitted photon energy, E_G_ is the band gap energy, E_A_ and E_D_ are the depths of the isolated donor and acceptor, respectively, R is the intra-pair separation, e is the electron charge, and K is the static dielectric constant^21^. It should be noted that the transition probability of D-A luminescence also depends on R. The transition probability (W(R)) as a function of R, using the Bohr radius (R_B_) of the donor, is given as W(R) = W_0_exp[-R/(R_B_/2)] where W_0_ is a constant^21^. Based on this relationship, the optical transition probability of the pair emission decreases with increasing R. The dependence of the luminescence spectrum on the electrical frequency is due pairs with larger R values saturating faster, resulting in their radiation ceasing to increase with increasing electrical frequency. Therefore, the number of high-energy (i.e., short wavelength) photons increases. Hence, the change in the operating frequency provides another method for colour tuning the ACEL devices. However, some specific materials properties, such as the co-activator elements present in the commercially available phosphors, were not available, making definitive explanations difficult. However, the frequency-modulated colour tuning in PDMS + ZnS can be explained considering the D-A pair luminescence model and optical transition probabilities under different frequencies.

The effect of the bottom layer of the device on the colour characteristics is also critical as the patterned region consists of two layers, namely, a PDMS + ZnS(G)/PDMS + ZnS(B) bilayer (inset of Fig. 1b). It could be anticipated that light from the bottom PDMS + ZnS(B) layer would influence the resultant colour performance, particularly at 1 kHz, as the layers emit green and blue EL with the largest colour difference. To confirm this effect, we fabricated a single-EML PDMS + ZnS(G) device and compared its spectrum with that of PDMS + ZnS(G/B) (Fig. 2c). An increase in the intensity over the range of 400–500 nm in the PDMS + ZnS(G/B) was observed, which was caused by leaky blue light from the bottom layer. Due to this spectral change, the CIE coordinates of (0.180, 0.450) shifted in a bluish direction to (0.167, 0.355) (Fig. 2b). Ideally, a larger colour difference in an ACEL device is desirable from the viewpoint of visibility. Although our developed device does not show optimised colour performance, we believe this can be improved by simply controlling the PDMS + ZnS mixture viscosity or the mesh hole size in the screen-printing fabric to increase the thickness of the patterned PDMS + ZnS(G) stripes. By doing this, we can expect a thinner bottom layer (PDMS + ZnS(B)) containing a small amount of ZnS(B), which would result in decreased light leakage. However, the present study does not contain an optimisation process because we believe that the performance demonstrated herein is sufficient as a proof-of-concept of patternable and widely colour-tunable EL devices.

**Figure 3:**
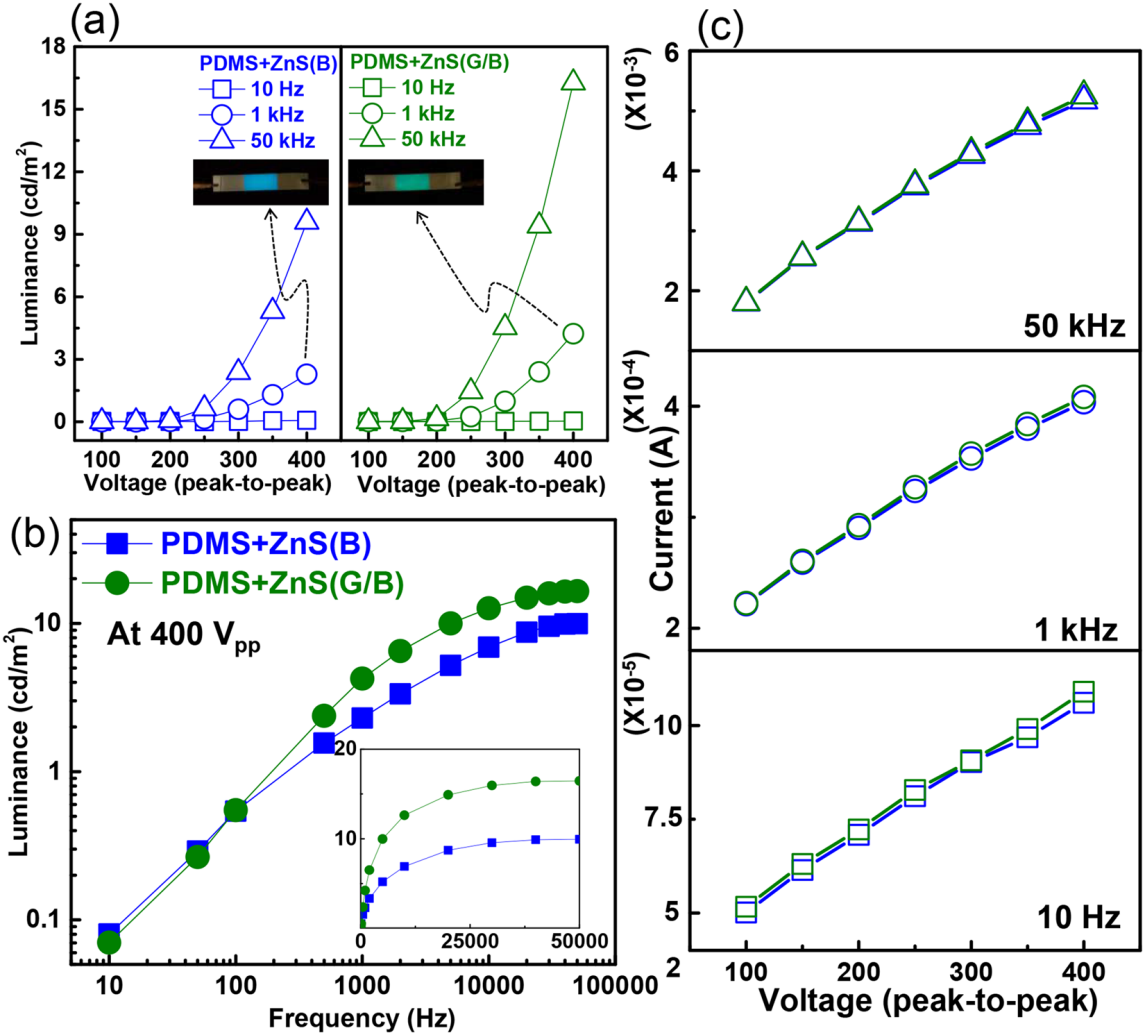
(**a**) Voltage-luminance, (**b**) frequency-luminance, and (**c**) AC current behaviour of PDMS + ZnS(B) and PDMS + ZnS(G/B) devices under different electrical frequency conditions (400 Vpp). Insets in (a): light-emitting ACEL images. Note that the data points of the two devices shown in (**c**) overlapped significantly due to their similar AC current levels.

From the viewpoint of device performance, it is also crucial to verify whether the screen-printed layer can realize comparable brightness performance. To confirm the independent electro-optical performance of the layers, we sliced a sample to create two ACEL devices, namely, PDMS + ZnS(G/B) and PDMS + ZnS(B) devices. Figure 3a shows the voltage-luminance relationship for each sample under various voltages. Both PDMS + ZnS(B) and PDMS + ZnS(G/B) showed increased luminance when the AC voltage increased due to the increased probability of electrons exciting luminescent centres. The applied high frequency induced high luminance as the concentration of free carriers in ZnS increased with increasing frequency during electroluminescence^29,30^. The higher luminance observed in PDMS + ZnS(G/B) compared to PDMS + ZnS(B) was due to the inherent characteristics of ZnS(G) as glass-based, single ZnS(G) devices also showed the same tendency (Fig. S2, Supplementary Information). We also measured luminance under various frequency conditions (Fig. 3b). As the electrical frequency increased, the luminance also increased. However, the luminance showed saturation behaviour because the optical transition probabilities of D-A pair luminescence were saturated and the radiation emission ceased to increase with increasing electrical frequency.

To confirm the difference in electrical properties between ZnS(B) and ZnS(G/B), we applied three representative frequencies to the two devices and measured very similar current values for both devices (Fig. 3c), indicating that there was no electrical problem in the bilayered structure produced by screen printing. Based on these electro-optical behaviours, which are also usually observed in conventional brittle ACEL, we confirmed that the present elastomer-based, screen-printed devices showed stable electro-optical performance.

Regarding the luminance of the devices, it is also important to comment on solutions to overcome the low brightness. In fact, the low dielectric constant of PDMS reduces its suitability for use in bright EL devices as most of the potential drop will occur within the matrix. A matrix with high dielectric constant will instead focus the electric field on the EL particles, which will increase charge separation within EL particles and thus the light emission^31^. Recently, this problem was overcome by adding BaTiO_3_ particles to the PDMS, resulting in the electric field being focused on the particles and improving light emission^7^. Another factor resulting in the low luminance of our device is the EML thickness (~100 μm in this case). Decreasing the EML thickness is expected to increase the EL brightness by increasing the electric field. Optimizing the screen-printing and moulding process to achieve thinner EMLs would enhance the luminance of the device. Therefore, in order to avoid weakening the electrical field at the location of the emitting particles, the layer has to be as thin as possible and have a dielectric constant as high as possible. Moreover, the transmittance of the AgNW-embedded PDMS film was only ~50% (at 550 nm)^10^, which was a major obstacle in achieving high luminance. The luminance of the present device is very low as important factors such as the dielectric constants, EML thickness, and transmittance of the electrodes, have not yet been optimized.

Another crucial factor for the proposed applications is the dependence of the luminance on frequency. Large differences in the luminance at different frequency ranges would be another critical obstacle for application of the present concept. Although, this study did not clarify these two important aspects for practical use, they would be interesting topics of further study. However, we believe that these problems can be resolved by optimising the materials and processes for producing the EML and AgNW electrodes.

**Figure 4:**
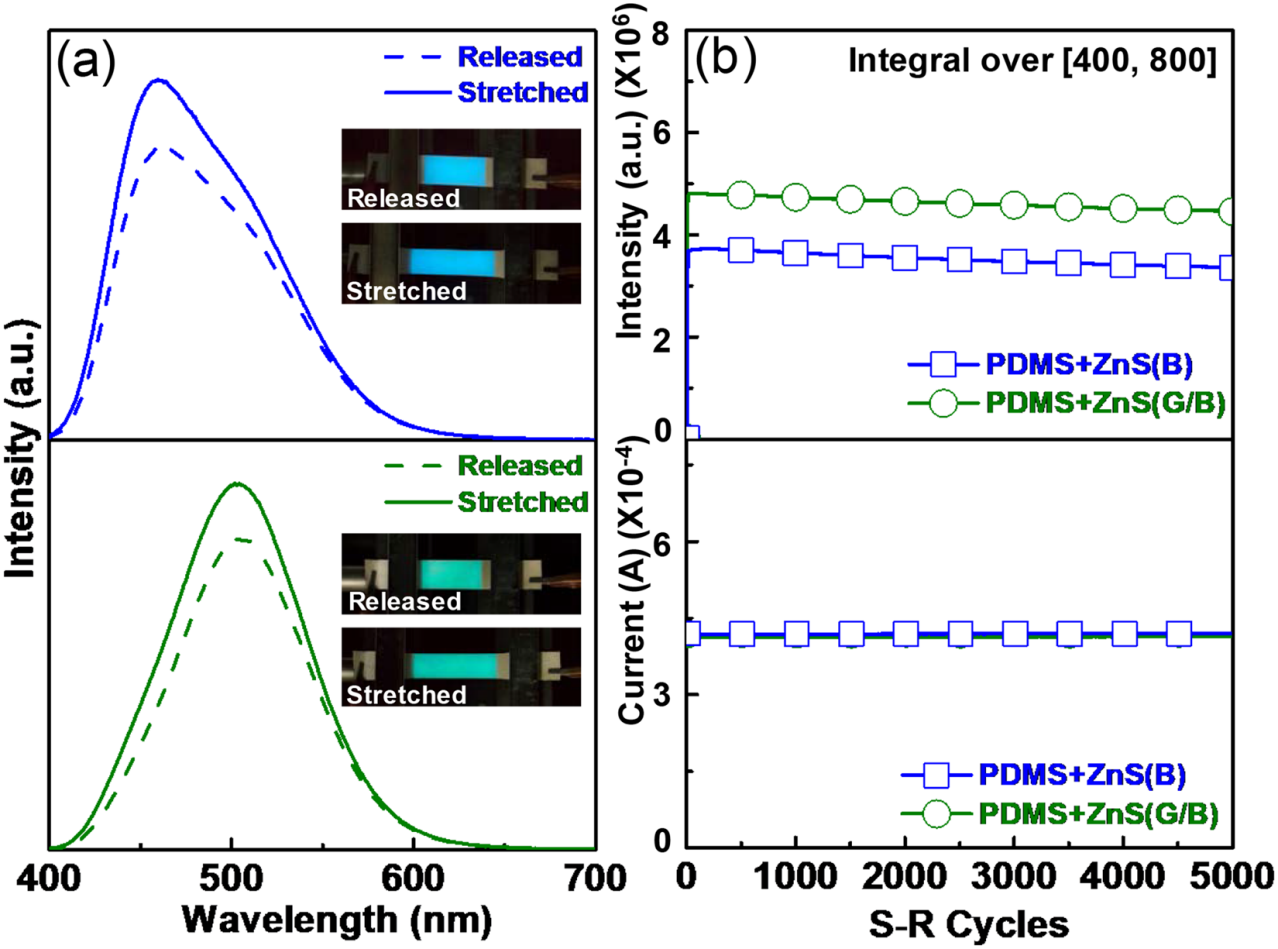
(**a**) EL spectra of PDMS + ZnS(B) (upper) and PDMS + ZnS(G/B) (bottom) devices under released and stretched conditions (40%). Insets: light-emitting ACEL images. (**b**) Time-dependent optical intensity (integrated values of EL spectra) (upper) and AC current behaviour (bottom) of two devices up to 5000 cycles of S-R motions.

Given that the developed screen-printed devices mainly consisted of PDMS and AgNW electrodes, stretchability and durability are important characteristics. When the devices were stretched under a constant applied voltage, increased luminous intensity was observed in both devices (Fig. 4a). As the sandwich structure in the AC field acts as a capacitor, increased EL intensity was observed under elongation. This increase was due to the decreased active layer thickness, which corresponded to an increase in the active layer capacitance. To analyse the potential applications of the present devices, we also examined the durability of the ACEL devices under stretch-release (S-R) motions (Fig. 4b). Because the AgNW-embedded PDMS electrodes were fabricated by a transfer process, the AgNWs were buried at the surface of the PDMS. The percolating network of AgNWs was also transferred and bonded to the cured PDMS after being peeled off the glass substrate^32^. Therefore, the AgNW film was located just below the PDMS surface, forming a conductive and stretchable layer (i.e., one of the top surfaces of the PDMS plates was an AgNW + PDMS composite). Since these electrodes were mechanically robust, durable EL behaviour was expected, even under repeated mechanical S-R motions. Actually, stable emission behaviour from the PDMS(AgNW)/PDMS + ZnS/(AgNW)PDMS structure was already reported in previous papers^4,11^. However, the stability of screen-printed EML devices has not yet been confirmed and it is unknown whether laminated EMLs with different compositions affect device stability during stretching. To confirm this effect, the device was stretched to a maximum elongation of 40% of its original length, and then allowed to recover to its original state; this was defined as one cycle of S-R motion. To avoid ML effects, we chose a slow S-R rate of 20 cycles per min (cpm). The EL intensity showed stable behaviour under continuous S-R motion up to 5000 cycles (Fig. 4b, upper). The weak degradation in luminous intensity (~8%) compared with the initial intensity was due to an inherent property of the material as the same tendency was observed without S-R motions (Fig. S3, Supplementary Information). Furthermore, the AC currents of the two devices were stable and almost overlapped, confirming that no electrical shortage during the S-R motion occurred (Fig. 4b, bottom). Based on these results, we confirmed that the screen-printed elastomer-based ACEL device had stable characteristics, even under repeated mechanical deformation.

**Figure 5:**
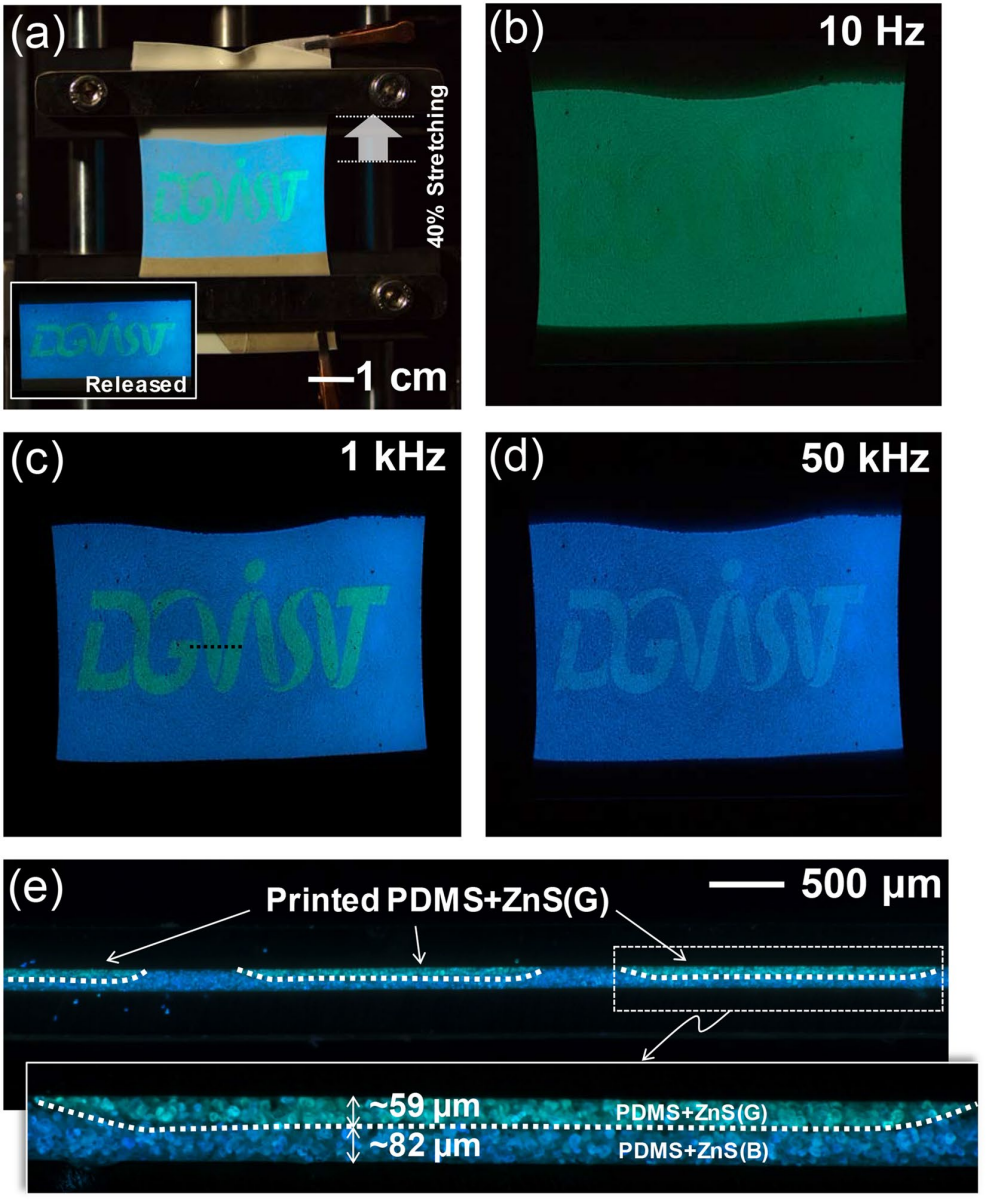
(**a**) Photograph of light-emitting logo-patterned ACEL device at the elongated condition (40%). Inset: light-emitting image under the released condition. Photographs of light-emitting images under electrical frequencies of (**b**) 10 Hz, (**c**) 1 kHz, and (**d**) 50 kHz at 400 Vpp. (**e**) Cross-sectional view of a sliced ACEL device (dotted line in (**c**)). Optical microscope images were taken under UV (365 nm) irradiation.

Based on our findings, we demonstrated herein a unique light-emitting device application using a colour-tuning concept with a screen-printed PDMS + ZnS device by controlling the applied electrical frequency. A pattern writing the word “DGIST” was printed from PDMS + ZnS(G) on a AgNW-embedded PDMS substrate using a conventional screen-printing method. Then, a PDMS + ZnS(B) mixture was sandwiched between two AgNW-embedded PDMS plates. Except for the DGIST pattern, the entire fabrication process was the same as that shown in Fig. 1a. In this case, the patterned logo consisted of PDMS + ZnS(G/B), while the background was PDMS + ZnS(B). To highlight the device stretchability, the test was performed under the condition of 40% elongated (Fig. 5a). Figure 5b–d show the colour tunability of the patterned device under different electrical frequency conditions. At low frequency (10 Hz), the DGIST logo was not obvious, as both PDMS + ZnS(G/B) and PDMS + ZnS(B) emitted green luminescence with a similar frequency (Fig. 5b). However, at 1 kHz, the background colour changed to blue, while the DGIST pattern still emitted green EL (Fig. 5c). The large colour difference between PDMS + ZnS(G/B) and PDMS + ZnS(B) allowed clear visualization of the patterned image. When we increase the frequency to 50 kHz, both colours changed to blue. Although the colours changed, the letters were still distinguishable as colour and luminance discrepancies existed to some extent (Figs 2b and 3a). Figure 5e shows a cross-sectional view of the patterned region, indicated by the dotted lines in Fig. 5c. The screen-printed PDMS + ZnS(G) was well laminated on PDMS + ZnS(B) without intermixing, which resulted in a clear patterned image of the logo.

**Figure 6:**
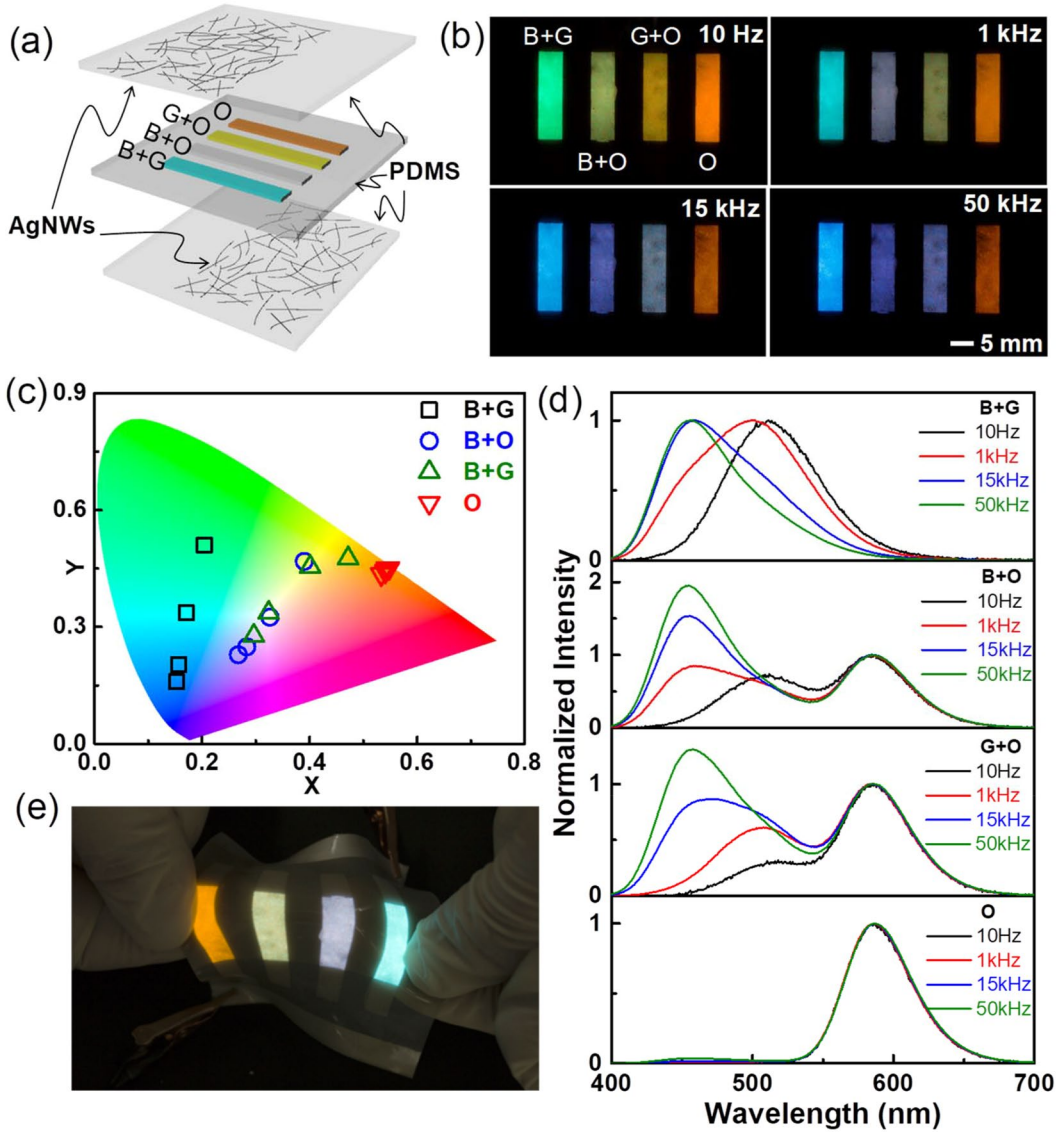
(**a**) Schematic of the ACEL device with four screen-printed stripes. (**b**) Photographs of the four light-emitting stripes at different electrical frequencies (10 Hz, 1 kHz, 15 kHz, and 50 kHz) at 400 Vpp. (**c**) The CIE coordinate (*x, y*) values and (**d**) EL spectra of each stripe normalized to the orange peak under various frequency conditions. (**e**) Photograph of the manually stretched light-emitting ACEL device (400 Vpp; 1 kHz).

Next, we consider the widely colour-tunable characteristics of the patterned device. It should be noted that mixing of multi-colour-emitting phosphors would allow a broader colour-tuning range. To demonstrate this, here we employed an orange-emitting phosphor (ZnS:Cu,Mn) hereinafter referred to as ZnS(O). Note that the colour of ZnS(O) is insensitive to the electrical frequency as orange emission from ZnS(O) originates from a radiative energy transfer process in which Cu behaves as a sensitizer. By mixing ZnS(O) with ZnS(B) or ZnS(G), we observed that the colour can be widely tuned. In this case, the CIE coordinates in colour space changed with an arcing shape due to a mixed effect of the fixed coordinates of ZnS(O) and the bluish shift of ZnS(B)/ZnS(G) in response to frequency modulation. To prove this concept, we fabricated four stripes on a screen-printed ACEL device with different mixing conditions; PDMS + [ZnS(B) + ZnS(G)], PDMS + [ZnS(B) + ZnS(O)], PDMS + [ZnS(G) + ZnS(O)], and PDMS + ZnS(O) (Fig. 6a). Hereinafter, these stripes are referred to as B + G, B + O, G + O, and O, with compositions of 5:5(B:G), 2.5:7.5(B:O), 1.5:8.5(G:O), and pure O, respectively. Four photos (Fig. 6b) and sets of CIE coordinates in colour space (Fig. 6c) clearly show the widely tunable colour characteristics of EL emitted from the four stripes. When we increased the electrical frequency from 10 Hz to 50 kHz, the colour of the B + G stripe changed from green to blue as both phosphors showed similar behaviour, as described in Fig. 2. However, when the ZnS(O) phosphors were mixed with ZnS(B) and ZnS(G), corresponding to the B + O and G + O stripes, the colours changed from light green or yellow to purple due to the contribution of the orange emission. In particular, we note that the B + O and G + O stripes appeared white, with CIE coordinates of (0.326, 0.325) and (0.324, 0.336) under 1 kHz and 15 kHz conditions, respectively, which were very close to the equal energy point (0.33, 0.33). As also shown in Fig. 6d, these colour changes were induced by an increase in the blue component in ZnS(B) and ZnS(G). We also note that the performance of the device was maintained during stretching (Fig. 6e). Together, these results demonstrate the widely tunable colour characteristics of stretchable, screen-printed, PDMS-based ACEL devices.

## Conclusions

In this study, we demonstrated widely colour-tunable characteristics of PDMS-based ACEL devices achieved by employing both a patterned EML and electrical frequency modulation. The electro-optical characteristics of the printed ACEL devices were similar to those of non-printed ones, indicating stable performance. The colour characteristics were also observed at increasing electrical frequencies, and we found a similar tendency for a bluish shift in both blue (PDMS + ZnS(B)) and green (PDMS + ZnS(G)) devices, but with different sensitivities to the applied frequency. Based on these results, we fabricated a logo-patterned ACEL device to demonstrate the colour variation effect. When increasing the electrical frequency, the logo and background showed similar green (10 Hz) and blue (50 kHz) colours, while a distinct colour difference was observed at 1 kHz. Notably, this interesting feature could be enhanced by mixing multi-coloured phosphors, which showed widely tunable colour performance. It would be very attractive if a light source could generate various colourful images simply in response to electrical frequency modulation, thereby creating a novel multi-colour functional display. We believe that our demonstrated method could be applicable for manipulating broader colour expression and that it will easily find roles in a broad range of applications involving stretchable devices.

## Methods

### ACEL device fabrication

The detailed fabrication process is illustrated in Fig. S1 (Supplementary Information). Briefly, the ACEL device comprised three layers, the EML sandwiched between two AgNW-embedded PDMS layers, which is an EL-emissive stretchable structure. First, AgNWs dispersed in ethanol (AgNWs-40, ACS Material) were spin-coated onto a poly(methyl methacrylate) (PMMA)-coated glass substrate. The thickness of the PMMA layer was approximately 200 nm. Spin-coating of the AgNWs was undertaken at 300 and 100 rpm for the upper and bottom plates, respectively, followed by drying at 100 °C for 1 min. Then, the two AgNW-embedded PDMS plates (approximately 300 μm in thickness) were prepared using a transfer process. The transmittance spectra and photographs of the AgNW-embedded plates have been previously reported, which indicated higher transmittance of the upper layer due to the high spin-coating speed (300 rpm)^10^. The higher transmittance in the upper layer was intentionally designed as the EL light transmits the upper layer. For the EL-emitting layer, liquid PDMS (ELASTOSIL RT601, Wacker) with a curing agent at a weight ratio of 9:1 was mixed with ZnS(G) phosphors (GG 45, Global Tungsten & Powders Corp.) at a 7:3 weight ratio. Next, the PDMS + ZnS(G) mixture was screen-printed on top of the AgNW-embedded PDMS. This technique involved pressing the mixture through a mesh with a previously designed pattern using a rubber squeegee. After subsequent curing at 70 °C for 35 min, PDMS mixed with ZnS(B) (GG65, Global Tungsten & Powders Corp.) at a 7:3 weight ratio was subsequently sandwiched between two AgNW-embedded PDMS plates; one of the plates contained cured PDMS + ZnS(G) stripes, and the device was then cured at 70 °C for 35 min. To prepare the widely tunable patterned stripes (Fig. 6), ZnS(O) (GG13, Global Tungsten & Powders Corp.) was mixed with ZnS(B) or ZnS(G) prior to mixing with PDMS.

### Electro-optical characterization

The EL spectra were measured by applying a square-wave voltage of 400 Vpp (peak-to-peak voltage) at various frequencies produced using a function generator (DG4062, RIGOL) with a voltage amplifier (HA-405, PINTEK). The AC current was measured using a current meter (3706 A, Keithley), and the light emission was observed using a spectrometer (QEPro, Ocean Optics, Inc.) combined with a vertically aligned optical fibre equipped with a collimating lens. To measure the luminance, a spectroradiometer (PR-670, Photo Research Inc.) was used. The CIE coordinates were calculated from the measured EL spectra. Photographs were taken with a Canon EOS 70D using proper manual conditions to visualize the images regardless of their luminance.

### Durability tests

Time-dependent optical intensity values (Fig. 4b, upper) obtained by integrating the spectral intensity from 400 to 800 nm were recorded every 3 s. In addition to time-dependent data, all EL spectra were recorded using a 1 s integrating time. The AC current (Fig. 4b, bottom) values were obtained by averaging data points over each 3-s period (30 points). The CIE coordinates were calculated from the measured EL data.

### Data availability

The datasets generated during and/or analysed during the current study are available from the corresponding author on reasonable request.

## Electronic supplementary material

Supplementary Information
