# Supplementary material for: Patternable and Widely Colour-Tunable Elastomer-Based Electroluminescent Devices
Source: Sci Rep. 2018 Feb 20;8:3331. doi: 10.1038/s41598-018-21726-x (PMC5820259; doi:10.1038/s41598-018-21726-x)
Supplement: Supplementary file 1 — Supplementary Information [file 41598_2018_21726_MOESM1_ESM.pdf]

Supplementary Information

Patternable and Widely Colour-Tunable Elastomer-Based Electroluminescent Devices

Seongkyu Song,[1] Hyunseok Shim,[2] Sang Kyoo Lim,[1] and Soon Moon Jeong[1]*

[1]Smart Textile Convergence Research Group, DGIST, Daegu 42988, Republic of Korea
[2]Intelligent Devices and Systems Research Group, DGIST, Daegu 42988, Republic of Korea

*Correspondence and requests for materials should be addressed to S.M.J. (E-mail: smjeong@dgist.ac.kr)

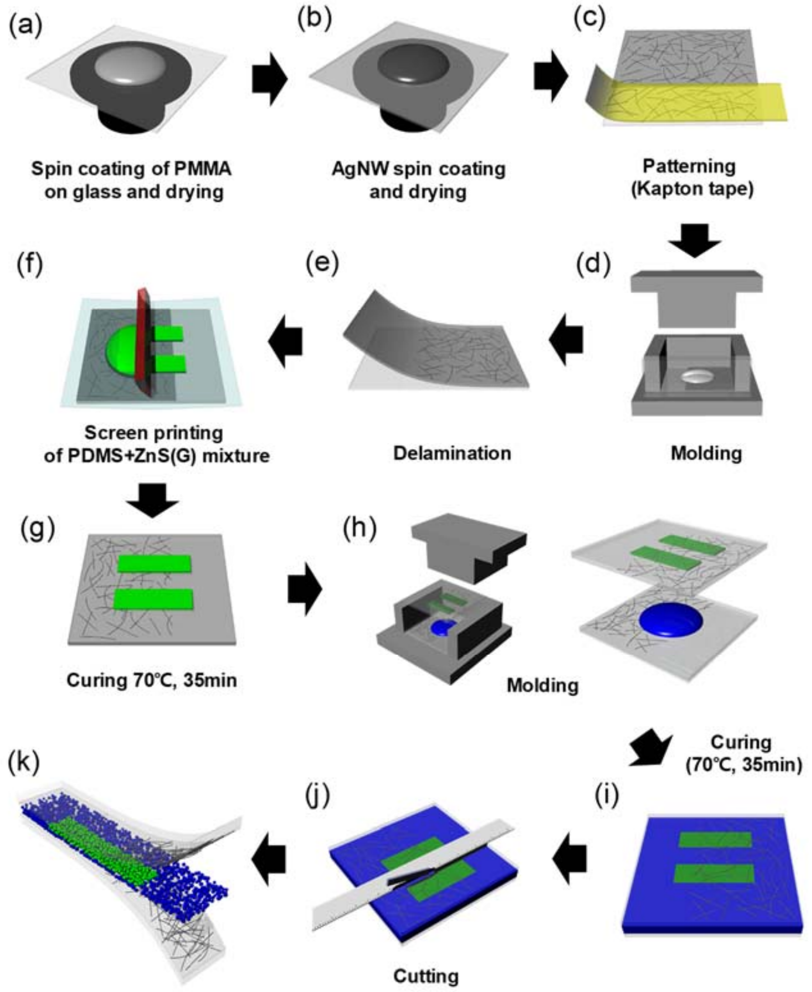

Figure S1. Schematic of the ACEL device fabrication process. (a,b) The AgNW solution (dispersed in ethanol) was spin-coated onto the PMMA (200 nm)-coated glass at 100 and 300 rpm for the bottom and top electrodes, respectively. After evaporating the ethanol at 100°C for 1 min, (c) a part of the AgNWs was removed with tape to form patterned electrodes. (d,e) Then, the PDMS solution was dropped onto the AgNW-coated glass and subsequently cured using a moulding process in an oven. (f,g) The PDMS+ZnS(G) mixture was screen-printed on AgNW-embedded PDMS and then cured. (h,i) The PDMS+ZnS(B) mixture was sandwiched between two AgNW-embedded PDMS plates (one of the plates contained cured PDMS+ZnS(G) stripes) and then cured. (j,k) The film was cut into the desired sample dimensions.

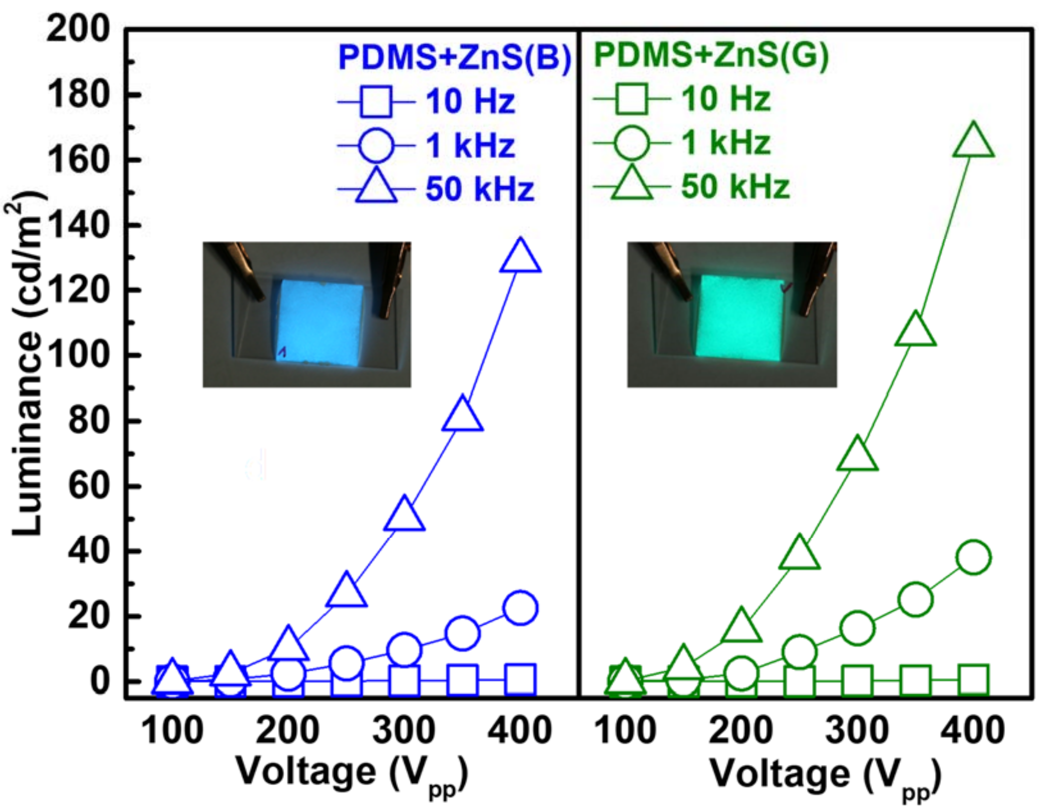

Figure S2. (a) Voltage-luminance curves of the PDMS+ZnS(B) and PDMS+ZnS(G) devices sandwiched between ITO-coated glass substrates.

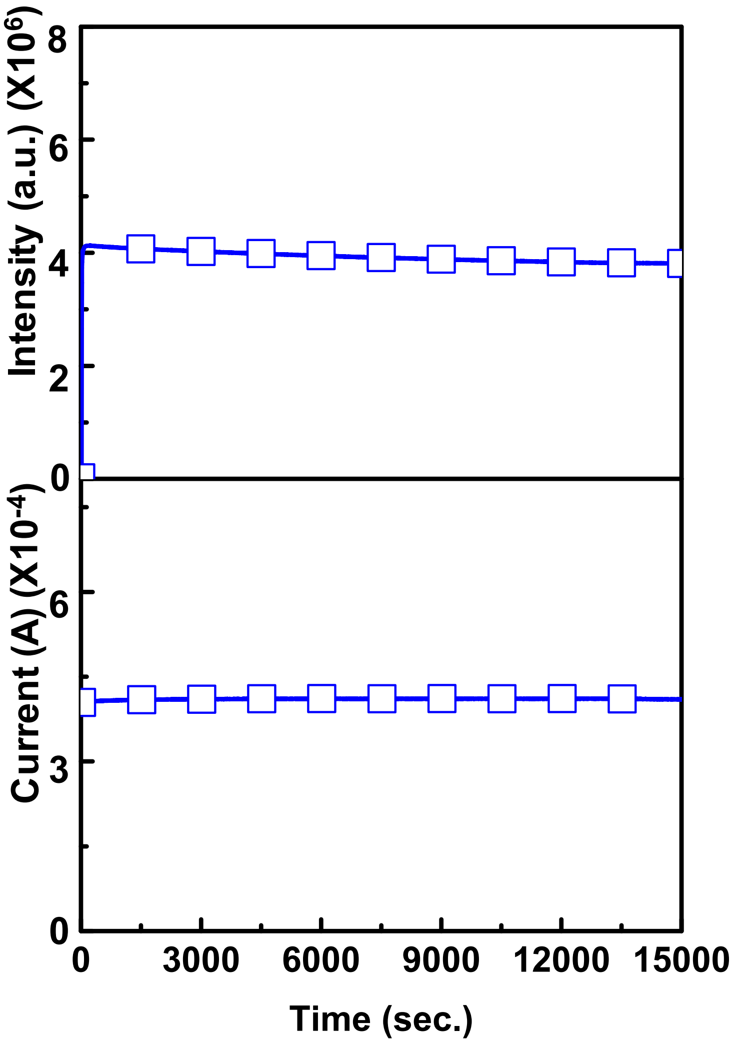

Figure S3. Time-dependent optical intensity (integrated values of EL spectra) (upper) and AC current behaviours (bottom) of a PDMS+ZnS(B) device without S-R motions. Note that the measurement time (15000 s) was set to be equal to that of the 5000 cycles of S-R motions shown in Figure 4b.
